# Standard operating procedures: therapeutic hypothermia in CPR and post-resuscitation care

**DOI:** 10.1186/cc11262

**Published:** 2012-06-07

**Authors:** Markus J Foedisch, Andreas Viehoefer

**Affiliations:** 1 Department of Anesthesia and Intensive Care Medicine, Evagelische Kliniken Bonn, Germany

## 

After two randomised studies published in 2002 [1,2] mild therapeutic hypothermia treatment was internationally recommended as early and efficious treatment for comatose survivors after cardiac arrest (CA) not only with ventricular fibrillation, but also for patients suffering from CA presenting with other initial rhythms (asystole, PEA) and different underlying causes. Therapeutic hypothermia has been shown in these investigations to improve not only survival significantly after CA but especially the neurologic outcome after different courses of cooling treatment. Nevertheless the in-hospital mortality of those patients remained high.

While several prehospital or pre-CPR factors contributing to the patients' outcome are well known and implemented in the BLS and ACLS guidelines, only little is known about the kind and impact of in-hospital contributing factors worsening the chance of surviving the event with good neurological function. After return of spontaneous circulation, major cardiovascular and haemodynamic disorders are widely common and associated with a high rate of deaths within the first 24 hours after CPR. Sufficient post-resuscitation therapy has to include optimal treatment strategies of the cardiovascular and metabolic system, adaequate ventilation support and strategies of neuroprotection [3]. In patients surviving with a favourable outcome, haemodynamic and respiratory disorders tend to normalise within the first 24 hours after ROSC.

Several factors of hospital care are obviously important for survival of post-CA patients. Observational investigations done in Norway and Sweden detected severe differences in outcome of patients admitted to hospital with ROSC after out-of-hospital CA presenting survival rates between 33 to 56% and 14 to 42% respectively [4-6]. There were no significant differences in the prehospital management of those patients, but in-hospital factors like blood glucose levels, seizures, body temperature and laboratory changes could be related to outcome. A similar cohort study using a multicentre clinical ICU registry in the United States enrolled 4,674 patients from 39 hospitals covering a 4-year hiatus showed the same interhospital variability in survival with an unadjusted mortality ranging from 41 to 81%. Those patients treated in centres with higher case volumes were significantly less likely to die in-hospital after ROSC independent of the location of the CA. As it was not possible to differentiate the effect of specific therapies and interventions on survival in the post-CA period, the results underlined the need for additional research to define optimal post-cardiac treatment strategies. The data underlined not only the volume-outcome relationship but also the necessity of implementing standardised guidelines for optimal post-CA care in specialised centres.

Based on this evidence a prospective observational study was performed in patients admitted to hospital after regaining ROSC and treated using a standardised treatment protocol including instant onset of therapeutic hypothermia, early reperfusion treatment with PCI, and protocol-based early-goal-directed therapy to restore adaequate arterial blood flow in the reperfusion period [7]. The observational group from the interventional period was compared with controls from an earlier period in the same hospital. There were not only major differences in survival but also in the quality of neurologic outcome. After implementation of the standardised treatment protocol, survival improved from 31% to 56% in the interventional period, 56% of the patients showed a favourable neurologic outcome (26% in the control period) at hospital discharge and were still alive after 1 year. With no changes in the algorithm of prehospital care in the years of the investigation, post-resuscitation care appeared to have a major effect on improving not only survival but also the neurologic outcome after successful CPR.

Despite the fact that the level of evidence for many of the treatment strategies with the exception of therapeutic hypothermia in post-resuscitation care is weak, the quality of care after admission to the ICU or ED seems to be a somewhat missing link in the chain of survival. The post-resuscitation phase is associated with a sepsis-like syndrome [8] of unknown time course causing or even intensifying global ischaemic brain damage and dysfunctional heart disease. Treatment of these disorders is the main challenge after ROSC, but implementation of such strategies is often slow and in a heterogeneous manner causing a widely variable state of post-resuscitation care.

Many factors (Table 1) may contribute to this phenomenon and show the complexity of treating patients after ROSC. This underlines the necessity of using protocol-driven care in those patients to help physicians and nurses to raise the level for the number of patients receiving standard therapy. It is obvious that such protocols have to be adapted to local hospital specialities and logistic factors.

**Table 1:** Inhospital factors influencing outcome of CA patients

Lack of implementation of therapeutic hypothermia and temperature management
Missing standard operating procedures/protocols for post-resuscitation care
Time lapse from ROSC to start of interventional phase
Treated case volumes of CA patients
Training and experience of personnel
Inadequate post-arrest treatment decisions

In our hospital an early algorithm for therapeutic hypothermia based on the standards used during the HACA trial [1] was designed and implemented immediately after enrolling patients for that European multicentre study in 2001. All patients being successfully resuscitated after CA independent from localisation, initial rhythm and type of the event were treated by therapeutic hypothermia and enrolled in our own database (CoolBrain Registry Bonn) including EMS data, course and technique of cooling and following temperature management, neurologic outcome at discharge and in a 1-year follow-up. Shortly after implementing the cooling protocol a special algorithm for general post-resuscitation care including therapeutic hypothermia and focusing on an early goal-directed approach to cardiac function, normoventilation, seizure treatment and strict avoidance of high blood glucose levels was designed and enabled physicians and nurses how to monitor and treat those patients. Baseline data of heart and brain function using invasive cardiac output monitoring and brain damage markers were included in the database as well. Both protocols and order sets are actualised to new guidelines and therapeutic standards based on actual science on a regular basis.

Unfortunately there is only limited access to outcome data of our patients treated before implementation of these SOP protocols, so the data from the CoolBrain Registry are only observational and cannot be compared with a control group before using protocol-driven therapeutic standards. Neurologic outcome was not recorded before implementing therapeutic hypothermia as standard care in 2001; survival rates after OHCA were recorded between 1990 and 2000 as lower than 20%.

Data enrolled from March 2001 to December 2011 (*n *= 276) presented a general survival rate of 51% independent of origin, initial rhythm and localisation of the cardiac arrest. Thirty-nine per cent of those patients showed a favourable neurologic outcome (CPC 0.5), 12% severe neurologic disability. In the group of survivors, 69.5% of patients with ventricular fibrillation as the initial rhythm showed an excellent neurologic performance (CPC 1) and 35% of patients presenting with asystole/PEA as well at hospital discharge.

Despite missing a control group before implementing therapeutic hypothermia and standardised treatment protocols for post-resuscitation care, these data show the effect of protocol-based treatment especially on neurologic outcome of those patients. This underlines that more attention has to be focused on optimising and standardising post-resuscitation care to improve survival and neurologic outcome. Treatment of patients with CA does not end with ROSC; despite the fact that there is only weak scientific level of evidence for some singular strategies of post-CA treatment, the combination of an aggressive multifactorial therapeutic approach including temperature management significantly improves outcome. Therefore further clinical trials of other post-resuscitation therapies seem to be essential.
